# Reduced Graphene Oxide/Waste-Derived TiO_2_ Composite Membranes: Preliminary Study of a New Material for Hybrid Wastewater Treatment

**DOI:** 10.3390/nano13061043

**Published:** 2023-03-14

**Authors:** Andrea Basso Peressut, Cinzia Cristiani, Giovanni Dotelli, Anna Dotti, Saverio Latorrata, Ana Bahamonde, Antonio Gascó, Daphne Hermosilla, Riccardo Balzarotti

**Affiliations:** 1 Department of Chemistry, Materials and Chemical Engineering “Giulio Natta”, Politecnico di Milano, Piazza Leonardo Da Vinci 32, 20133 Milan, Italy; 2 Instituto de Catálisis y Petroleoquímica, Consejo Superior de Investigaciones Científicas, Calle de Marie Curie 2, 28049 Madrid, Spain; 3 Departamento de Ingeniería y Gestión Forestal y Ambiental, Universidad Politécnica de Madrid, Calle de José Antonio Novais 10, 28040 Madrid, Spain; 4 Department of Innovative Technologies, University of Applied Sciences and Arts of Southern Switzerland, Via la Santa 1, 6962 Lugano, Switzerland

**Keywords:** reduced graphene oxide, titanium dioxide, tionite, wastewater treatment, self-assembling, metal ions, filtration, organic pollutants, photodegradation, membranes

## Abstract

This work reports the preliminary results of the development of composite self-assembling membranes obtained by the combination of reduced graphene oxide (rGO) with commercial Degussa P25 titanium dioxide (TiO_2_). The purpose is to demonstrate the possibility of combining, in the same self-standing material, the capability to treat wastewater containing both inorganic and organic pollutants by exploiting the established ability of rGO to capture metal ions together with that of TiO_2_ to degrade organic substances. Moreover, this study also investigates the potential photocatalytic properties of tionite (TIO), to demonstrate the feasibility of replacing commercial TiO_2_ with such waste-derived TiO_2_-containing material, fulfilling a circular economy approach. Thus, rGO–TiO_2_ and rGO–TIO composite membranes, 1:1 by weight, were prepared and characterized by SEM-EDX, XRD, thermogravimetry, as well as by Raman and UV-Vis spectroscopies to verify the effective and homogeneous integration of the two components. Then, they were tested towards 3-mg L^−1^ aqueous synthetic solutions of Fe^3+^ and Cu^2+^ ions to evaluate their metal adsorption ability, with values of the order of 0.1–0.2 mmol g_membrane_^−1^, comparable or even slightly higher than those of pristine rGO. Finally, the ability of the composites to degrade a common organic pesticide, i.e., Imidacloprid^®^, was assessed in preliminary photocatalysis experiments, in which maximum degradation efficiencies of 25% (after 3 h) for rGO–TiO_2_ and of 21% (after 1 h) for rGO–TIO were found. The result of tionite-containing membranes is particularly promising and worthy of further investigation, given that the anatase content of tionite is roughly 1/6 of the one in commercial TiO_2_.

## 1. Introduction

The availability of potable water is one of the main issues to be solved in the near future. According to the World Water Development Report of the United Nations [1], a country experiences “water stress” when its annual water resources are below 1700 m^3^ per inhabitant. Climate changes and indiscriminate use of fresh water sources make this problem particularly compelling, mainly due to the growing water demand by all sectors, combined with the increasing variation in rainfall patterns. For instance, droughts and water scarcity are reported for a wide range of river basin districts across Europe, even in geographical areas not commonly subject to these phenomena [1,2]. Moreover, water is heavily exploited in several industrial sectors, such as agriculture, farming, pharmaceuticals, and manufacturing [2,3].

In particular, chemical pollution is a critical point for community water; for instance, one-fourth of the European surface water is below the quality target indicated by the 2000/60 Water Framework Directive [3], due to the presence of fertilizers, pesticides, and polluting sediments, as well as heavy metals like mercury, cadmium, nickel, and lead. Besides the elements mentioned above, iron, manganese, silver, copper, and aluminum can be added to the list of dangerous substances whose release in the environment must be avoided. In this scenario, the reduction of water contamination, from both inorganic and organic species, has come under intensive research, both at the academic and at the industrial level. The challenge is to find easy, efficient, and cost-effective technological solutions that can drastically reduce the content of pollution and undesirable species in wastewater [4,5].

Adsorption is one of the most promising methods of wastewater treatment, thanks to renowned effectiveness, affordability, and technical feasibility. Several adsorbents are used for micro- and ultra-filtration—namely, activated carbon [6], pristine and modified clays [7], activated silico-aluminates, and polymeric materials [8,9]. However, the removal of heavy metal ions from wastewater requires unconventional nanofiltration or reverse-osmosis techniques. Several works demonstrated the suitability of various types of nanomaterials for such filtration modes [10,11,12]: carbon nanotubes (CNTs), zeolites, nanofibers, graphene, and graphene oxide (GO). Among them, GO and its derivatives proved worthy of further in-depth studies. In particular, reduced graphene oxide (rGO) exhibits similar properties to pristine graphene because of a heterogeneous structure that comprises a graphene-like basal plane where structural defects and areas containing oxidized chemical groups are present [13,14]. The adsorption capability of rGO membranes towards Fe^3+^, Cu^2+^, Ni^2+^, and Mn^2+^ metal ions in model mono- and multi-ionic solutions was already demonstrated [15]. Focusing on single-ion solutions, a large affinity between sorbent and metal ions was evidenced since capture efficiencies higher than 50% were found for all the tested species, with a much higher adsorption capability towards Fe^3+^ and Ni^2+^, whose efficiency even exceeded 90% [15].

On the other hand, the application of advanced oxidation processes (AOPs) in the treatment of a wide range of organic pollutants (OPs), such as pesticides or fertilizers, presents excellent removal efficiency [16]. Among AOPs, semiconductor-mediated photocatalysis has gained great significance due to its potential to mineralize, at ambient temperature and pressure, a wide range of recalcitrant OPs, resulting in their complete oxidation into harmless substances [17]. The materials designated to foster photocatalysis are metal oxide nanoparticles (NPs), with titanium dioxide (TiO_2_)—in particular, the anatase phase—being the material of choice [10,18,19]. The appeal of TiO_2_ is emphasized by the possibility to either mold stand-alone filters or to produce thin-film nanocomposite membranes when dispersed in an adequate matrix, usually polymeric or carbon-based, resulting in an adsorbent able to purify industrial water from both inorganic pollutants (IPs) and OPs [20,21,22]. Moreover, the energy needed to photoexcite the catalyst can be directly obtained from the sun [23]; Carbajo et al. [24] reported a successful degradation of a complex mixture of pesticides by using a TiO_2_-based catalyst in a solar pilot plant, bringing wastewater treatment towards a greener approach. Nevertheless, TiO_2_ suffers from a large bandgap, which favors electron-hole recombination and results in a poor light harvesting, limiting the quantum efficiency [20,25,26,27,28].

In light of the research discussed, the combination of rGO ability to retain IPs with TiO_2_ photocatalytic properties to degrade OPs is considered a good choice for wastewater treatment purposes, as it combines both photocatalytic and adsorption capabilities in the same composite material [14,25,26,27,29,30,31,32,33,34]. Specifically, graphene-based materials are reported to enhance the photodegradation performances of TiO_2_ by adsorbing the contaminants and holding them close to the TiO_2_ photocatalyst surface [33]. Moreover, the rGO capability to absorb light in the UV-Vis range can counteract the undesirable recombination processes taking place in TiO_2_ [28]. Many methods were proposed in the literature to produce nanocomposites with the required properties, such as hydrothermal reaction [25,26,32,35,36], thermal-hydrothermal synthesis [37], electrodeposition [31], and liquid phase deposition [27]. Types of GO reduction [38], materials characterization, and photocatalytic properties were investigated as well [29].

In the literature, the photocatalytic activity of nanocomposites is usually evaluated in slurry form [14,26,27,36], and few papers deal with obtaining formed devices [29,31]. The use of slurry catalysts is useful when considering studies on mass transfer, kinetics, and reaction mechanism. However, a catalyst in slurry form is hardly used in “in-field” applications such as, for instance, the treatment of largely polluted streams. Moreover, the application of nanocomposites in slurry form may result in nanoparticle agglomeration, causing a serious decrease in the photocatalytic performance [39]. Hence, catalyst forms other than slurry, i.e., immobilized catalysts or membranes, are more suitable in practice [31].

In this respect, the use of graphene-based compounds also allows taking advantage of the self-assembly capability of GO-based materials, resulting in the production of separation membranes with superior water flux and great potential for application in wastewater purification [26,27,38,39,40]. Indeed, rGO can find use as a component in binary, ternary, or quaternary composites, coupling its capability to give hierarchical architectures with enhanced mechanical resistance, charge transfer, and ion diffusion processes [13]. This synergistic combination not only maintains all the interesting characteristics of its individual components but also overcomes the serious drawbacks of each one, such as low adsorption towards pollutants and rapid recombination of photogenerated electrons [39]. Luna-Sanguino et al. [28,41] studied the application of new titania-reduced graphene oxide (TiO_2_–rGO) nanocomposites to assess the solar-assisted photodegradation of a complex mix of pesticides (Pyrimethanil^®^, Isoproturon^®^, Alachlor^®^, and Methomyl^®^). Two TiO_2_–rGO nanocomposites were prepared using a hydrothermal method from commercial TiO_2_ (P25 and Hombikat UV100, HBK). Complete removal of pesticides was achieved, and the HBK TiO_2_-reduced graphene oxide nanocomposite presented excellent photoefficiency, especially under a natural water matrix, because of its large, negatively charged surface area, where the recombination of electrons and holes is probably prevented.

This paper presents the development of new rGO–TiO_2_ nanocomposite membranes with the aim of demonstrating their possible application in solar-assisted photodegradation, opening the opportunity to extend the use of these hybrid photocatalysts to complex wastewater effluents. Reduced graphene oxide (rGO), achieved by an eco-friendly controlled reduction using L-ascorbic acid (L-AA), and Degussa P25 titanium dioxide (TiO_2_) are selected as principal components of the water treatment technology, based on the established ability of rGO to remove inorganic pollutants and on the well-known capability of titanium dioxide to degrade organic contaminants. The purpose is to combine, in the same self-standing object, the ability to capture metal ions and to photodegrade organic pollutants. Indeed, rGO–TiO_2_ systems are already reported as suitable to be applied to wastewater treatment, but no results are available on the combination of the previous functions in the same membrane-like material. Furthermore, membranes produced using waste materials, which are unconventional with respect to the current technology, are also proposed in this work with a view of circular economy, in order to verify the feasibility of the partial replacement of commercial TiO_2_. In particular, tionite, namely, the residue from the dissolution process of the slags resulting from TiO_2_ manufacturing, is studied in combination with rGO to test its applicability as a photocatalyst.

Hence, composite membranes based on rGO and Degussa P25 TiO_2_, or rGO and tionite were prepared and characterized by X-ray diffraction (XRD), thermogravimetry, scanning electron microscopy (SEM), Raman, UV-Vis, and energy-dispersive X-ray (EDX) spectroscopies. Then, their adsorption and photocatalytic behaviors were tested separately towards water-based solutions of either metal ions, namely, Fe^3+^ and Cu^2+^, or of an organic pesticide, i.e., Imidacloprid^®^. The ion capture ability of the membranes, as well as their photodegradation properties towards the organic molecule, was compared with the final goal of demonstrating, albeit preliminarily, the combined capability to potentially treat wastewater of complex composition via adsorption and photodegradation.

## 2. Materials and Methods

### 2.1. Materials

A commercial GO aqueous suspension (4 mg mL^−1^, particle size < 10 µm [42], supplied by Graphenea, San Sebastian, Spain) was used as a starting material to obtain rGO. Degussa P25 TiO_2_ (TiO_2_ in the following, used as pure oxide), L-ascorbic acid, Fe(NO_3_)_3_·9H_2_O, Cu(NO_3_)_2_·3H_2_O, and Imidacloprid^®^ were acquired from Sigma Aldrich (Milan, Italy). Tionite (TIO in the following) was provided by Opigeo S.r.L (Grisignano di Zocco, VI, Italy).

### 2.2. Membranes Preparation

Two different types of rGO-based membranes were produced: rGO–TiO_2_ and rGO–TIO, combining rGO with either Degussa P25 TiO_2_ or tionite. Pure rGO membranes were also prepared as a benchmark.

**Figure 1:**
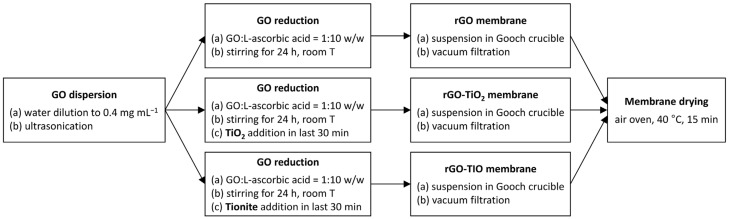
Flow diagram of the membranes production process.

A simple procedure (Figure 1) was adopted for all the membranes, implying the mixing of the components followed by the vacuum filtration of the obtained slurry [15,43].

In brief, the preparation comprises a four-step process:

(1) Dilution of 2 mL of the commercial GO aqueous suspension to 0.4 mg mL^−1^ and ultrasonication (Labsonic LBS1-6, Falc Instruments, Faenza, Italy).

(2) Reduction with L-AA for 24 h under stirring; a 1:10 weight ratio between GO and L-AA was selected [15]. In the case of rGO–TiO_2_ and rGO–TIO membranes, 8 mg of TiO_2_ or tionite, respectively, were added to the suspension 30 min before the end of the stirring time. For both composite membranes, a 1:1 weight ratio was set between the components.

(3) Pouring of the suspension into a Gooch crucible and vacuum filtration onto a PVDF filter (47-mm diameter, 0.45-µm pore size) by Merck Millipore (Burlington, MA, USA), until the formation of a self-standing membrane.

(4) Drying of the formed membranes in an oven at 40 °C for 15 min.

### 2.3. Materials and Membranes Characterization

Laser granulometry was conducted by means of CILAS 1180 (dispersion in water, CILAS, Orléans, France), according to the ISO 13320 standard and the technical report released in reference [44].

XRD analyses were performed by a Bruker D8 Advanced diffractometer (Milan, Italy) with the following conditions: Cu Kα radiation (0.154 nm), 2θ range = 2–60°, step size = 0.02°, and time per step = 12 s. Crystallite dimensions were calculated according to the Debye–Scherrer equation on the basis of the full width at half maximum (FWHM) of the XRD reflection [45].

Thermogravimetric (TG-DTG) measurements were conducted between room temperature and 1000 °C by using the EXSTAR 6000 TG/DTA 6300 by Seiko Instruments, Inc. (Chiba, Japan). The specimens were heated by a controlled ramp of 10 °C min^−1^ while exposed to an inert N_2_ atmosphere fluxed at 55 mL min^−1^.

Raman spectroscopy was carried out between 100 and 3300 cm^−1^ with a micro-Raman setup, consisting of the Jobin Yvon LabRAM HR800 spectrometer by HORIBA (Kyoto, Japan), equipped with a 50×-objective BX41 microscope by Olympus (Milan, Italy). A 632.8-nm helium–neon (He–Ne) laser, whose power was limited to 50 µW to minimize the possible heating and degradation of the investigated materials, was employed.

UV-Vis spectra were collected using a Jasco V-570 UV-Vis/NIR spectrophotometer (Jasco Europe, Milan, Italy) equipped with an integrating sphere diffuse reflectance accessory; the analysis was performed in the 250–750-nm range, and the scanning speed was set at 200 nm min^−1^.

The membranes, before and after their use, were also analyzed by scanning electron microscopy and energy dispersion X-ray spectroscopy (SEM-EDX), using a Zeiss EVO 50 EP (Zeiss, Jena, Germany) combined with a spectrometer Oxford INCA energy 2000 (Oxford Instruments, Abingdon, UK). The SEM-EDX equipment was operated at an electron high tension (EHT) voltage of about 20 kV, a current probe of 120 pA, and at high vacuum (about 10^−4^ Pa) to acquire images from both secondary and backscattered electrons. A semi-quantitative EDX investigation was performed on three independent areas (500× magnification) of each membrane sample by assessing the distribution of the detected species in elemental maps and by averaging the corresponding compositional information.

### 2.4. Adsorption Tests

In order to evaluate how the capture capability was influenced by the membrane composition, metal adsorption tests were performed by contacting each membrane with single-ion solutions of Fe^3+^ and Cu^2+^, as representative of common inorganic contaminants. Solutions of 3-mg L^−1^ ion concentration were prepared starting from either Fe(NO_3_)_3_·9H_2_O or Cu(NO_3_)_2_·3H_2_O, which correspond to metal salt concentrations of 21.7 mg L^−1^ or 11.4 mg L^−1^, respectively. The pH of the solutions was measured by a FiveEasy pH-meter equipped with a LE438 pH electrode (Mettler Toledo, Columbus, OH, United States). A qualitative prediction of the chemical equilibrium of the species available in solution, as a function of pH, was done with the Hydra-Medusa software [46].

Adsorption tests were performed by pouring 50 mL of each metal ion solution on the membranes. After 15 min of static contact, the solution was vacuum filtered and the filtrate subsequently recovered, as sketched in Figure S1 (Supplementary Materials).

The analysis of metal ion concentrations in the solutions, before and after the filtration step, was performed by means of inductively coupled plasma optical emission spectroscopy (ICP-OES), using a PerkinElmer OPTIMA 7000 DV spectrometer (PerkinElmer, Waltham, MA, United States). The average of three measurements is reported, and the estimated error is within 1%.

The specific amount of captured metal ions per gram of membrane was calculated as in Equation (1), starting from the difference between the concentrations of metal ions in solution, as determined by ICP-OES, before (C_i_, mg L^−1^) and after (C_f_, mg L^−1^) contact with the membranes:

(1) $$Q_{m}\left({\mathrm{mmol}\;g}_{\mathrm{membrane}}^{-1}\right)=\frac{\left(C_{i}-C_{f}\right)\cdot V_{1}}{{\mathrm{AW}}_{\mathrm{ion}}\cdot w_{\mathrm{membrane}}}$$

V_1_ (L) is the filtered volume of the mono-ionic solution (50 mL), AW_ion_ (mg mmol^−1^) the atomic weight of the adsorbed metal, and w_membrane_ (g) the average membrane weight.

### 2.5. Photocatalytic Activity

In order to evaluate the photodegradation capability of the membranes, preliminary photocatalytic tests were performed considering a 5-mg L^−1^ solution of a common pesticide, i.e., Imidacloprid^®^ (C_9_H_10_ClN_5_O_2_, 255.66 g mol^−1^, Figure S2).

The tests were carried out in a discontinuous-type batch photoreactor (Figure S3), equipped with an external irradiation provided by a 365-nm UV-A LED lamp, model BLS-13000-1 by Mightex (Toronto, ON, Canada). Each test was performed at a current intensity value of 250 mA and a power of 1.6 W, corresponding to an irradiance of 454 W m^−2^, measured by a UV-A CUV4 radiometer (Kipp & Zonen, Delft, The Netherlands). Atmospheric pressure and ambient temperature (20–25 °C), employing air oxygen as an oxidant agent, were considered as initial operating conditions.

In a typical test, 30 mL of the solution containing 5 mg L^−1^ of Imidacloprid^®^, at natural pH 6, were put in contact, for 30 min under dark conditions, with one of the rGO-based composite membranes to ensure the adsorption–desorption equilibrium of the pollutant on the surface of the photocatalyst. Afterwards, every photocatalytic run began by turning the LED lamp on (experiment time set at 0 h). Aliquots of the reactant solution were extracted at selected periods (0, 1, 2, 3, 4, 5 h) and filtered by PTFE syringe filters (25-mm diameter, 45-mm sieving size) by Teknokroma (Sant Cugat del Vallés, Spain) to monitor the performance of the reaction. The glass crystallizer that contained solution and membrane was kept under a constant, mild stirring of 80 rpm for the entire duration of the test (30 min under dark conditions, followed by 5 h of irradiation).

For comparison purposes, photolysis and adsorption tests were performed in the same experimental conditions in order to verify, respectively, the possible photodegradation of Imidacloprid^®^ in the absence of a photocatalyst and the amount of pollutant adsorbed by the nanocomposite membranes in the absence of irradiation.

Imidacloprid^®^ was identified and quantified by high-performance liquid chromatography (HPLC), model Azura by Knauer (Berlin, Germany), equipped with a photodiode array detector and an Ultrasep ES Pest 5-μm column (250 mm long, 3 mm in diameter).

The total organic carbon (TOC) was measured with an infrared TOC-V CSH analyzer by Shimadzu (Kyoto, Japan).

The ion chromatograph (IC) with a chemical suppression and conductivity detector, model 883 Basic IC plus by Metrohm (Herisau, Switzerland), was used to identify short-chain organic acids and cations. Short organic acids were identified with a Metrosep A supp 5-250 column (250 mm long, 4 mm in diameter) as the stationary phase, and 3.2-mM Na_2_CO_3_ and 1-mM NaHCO_3_ as the mobile phase. Cations were analyzed by means of a Metrosep C6-250 column (250 mm long, 4 mm in diameter) as the stationary phase and nitric/dipicolinic acid as the mobile phase.

The obtained results, averaged over two measurements with an estimated error lower than 1.5%, allowed assessing the photodegradation efficiency and to compute the specific amount of photodegraded Imidacloprid^®^ per gram of anatase (photoactive phase), according to Equation (2):

(2) $$Q_{a}\left({\mathrm{mmol}\;g}_{\mathrm{anatase}}^{-1}\right)=\frac{\eta_{\max,\mathrm{membrane}}\cdot C_{0}\cdot V_{2}}{{\mathrm{MW}}_{{\mathrm{Imidacloprid}}^{®}}\cdot w_{\mathrm{anatase}}}$$

Here, η_max,membrane_ is the maximum photodegradation efficiency of the membrane, C_0_ (5 mg L^−1^) the initial concentration of Imidacloprid^®^, V_2_ (L) the volume of the treated solution (30 mL), MW_Imidacloprid_^®^ (mg mmol^−1^) the molecular weight of Imidacloprid^®^, and w_anatase_ (g) is the amount of the anatase phase actually contained in rGO–TiO_2_ and rGO–TIO membranes, as estimated from XRD and chemical composition data.

## 3. Results and Discussion

### 3.1. Starting Materials Characterization

**Figure 2:**
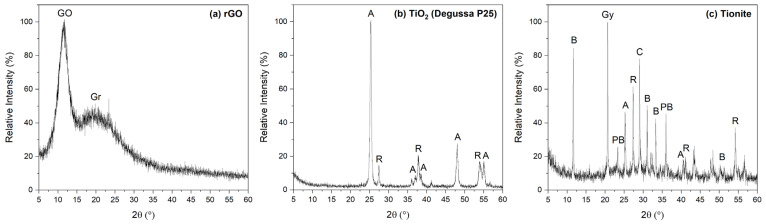
XRD patterns of (**a**) rGO, (**b**) Degussa P25 TiO_2_, and (**c**) tionite. In the figure, GO—graphene oxide, Gr—graphite, A—anatase, R—rutile, C—calcite, Gy—gypsum, B—bassanite, and PB—pseudo-brookite.

The XRD pattern of the pristine rGO membrane (Figure 2a) shows a strong, broad reflection at about 11° 2θ, corresponding to an interlayer distance of 0.80 nm, more than twice that of the typical one of graphite (d = 0.34 nm). This result, combined with the additional broad shoulder centered at about 22°, is consistent with a partial reduction of GO. This process induces the removal of oxygen-containing groups and the introduction of defects, causing the coexistence between still-oxidized regions and domains characterized by partial graphitization and structural amorphization [15,25,32].

Commercial TiO_2_ (Degussa P25) consists of a pure, white, fine powder with particle dimensions of 0.68, 2.93, and 6.41 µm (Figure S4a in Supplementary Materials). In the diffractogram (Figure 2b), both anatase (A, in the following) [JCPDS card no. 21-1272] and rutile (R, in the following) [JCPDS card no. 21-1276] phases are present; anatase is the main phase (83%), which corresponds to an A/R ratio equal to 4.8.

Tionite is a fine red-brown powder with particle dimensions of 1.25, 22.42, and 62.37 μm (Figure S4b), about one order of magnitude greater than Degussa P25 TiO_2_. It is a byproduct of TiO_2_ preparation, formed during acid etching of ilmenite (FeTiO_3_), i.e., the starting mineral for TiO_2_ production [47]. Due to its origin, it is characterized by a complex phase composition (Figure 2c). By combining the elemental composition (Table S1, given by the supplier Opigeo S.r.L) and the diffractogram, it is possible to tentatively identify the following main phases: calcite (CaCO_3_) at about 30° 2θ [JCPDS card no. 5-586]; gypsum (CaSO_4_·2H_2_O) at about 21° 2θ [JCPDS card no. 01-070-0982]; bassanite (CaSO_4_·0.5H_2_O) at about 12° 2θ, between 30° and 35° 2θ, and between 40° and 50° 2θ [JCPDS card no. 00-041-0224]; pseudo-brookite (Fe_2_TiO_5_) at about 23° and 35° 2θ [JCPDS card no. 009-0182], and TiO_2_ rutile and anatase phases. TiO_2_ content is about 30.1% of the total, and rutile is the main phase (57%), with A/R = 0.75; thus, the actual amount of anatase in tionite is about 13%.

**Figure 3:**
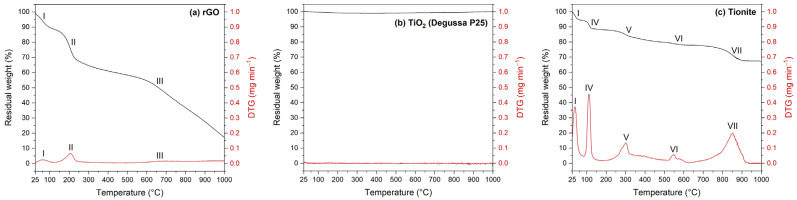
Thermogravimetric plots of (**a**) rGO, (**b**) Degussa P25 TiO_2_, and (**c**) tionite.

The results of thermal analysis are reported in Figure 3. Pristine rGO (Figure 3a) exhibits three fundamental weight losses: the first one (I), about 11.4%, can be ascribed to the removal of physically adsorbed water below 100 °C; the second one (II) results in a 29.9% weight decrease between 150 and 250 °C, and may correspond to the degradation of residual oxygenated moieties; the third one (III) can be associated with the final decomposition of the graphitic framework, taking place above 600 °C and causing a gradual weight drop of about 41.8% [15,31].

As expected, no evident loss was recorded for Degussa P25 TiO_2_ (Figure 3b) in the analyzed temperature range [30,37]. On the other hand, tionite (Figure 3c) seems to be characterized by a more complicated set of thermal phenomena, which are a consequence of the heterogenous composition of the material. The overall weight loss is limited to about 32.5% in the examined range and can be divided among five contributions. The first two, (I) and (IV), both correspond to a 5.9% loss; they can be ascribed to the removal of adsorbed humidity (below 50 °C) and of water chemically bound to ferrous sulfate multi-hydrate (FeSO_4_·*x*H_2_O, *x* = 4, 6, 7, at about 111 °C), respectively. The third contribution (V), at about 299 °C (8.5% loss), may represent the dehydration of ferrous sulfate monohydrate (FeSO_4_·H_2_O), which is then decomposed, together with ferric sulfate (Fe_2_(SO_4_)_3_), in the fourth stage (VI) at about 547 °C (1.7% loss) [48,49,50,51]. The last thermal event (VII) produces the largest weight loss (10.5%) above 700 °C and can be attributed to the decomposition of calcite (CaCO_3_) [52,53].

The results of Raman spectroscopy are displayed in Figure S5. The Raman spectrum of the rGO membrane exhibits its characteristic D and G bands. The former, centered at about 1333 cm^−1^, accounts for the structural disorder of the material due to the residual presence of oxygenated functionalities and to the defects introduced by the reduction process [27,34]. The latter, located at about 1604 cm^−1^, can be ascribed to the π-conjugated carbon atoms belonging to graphitic domains [35,37]. Its blue-shift with respect to the typical position of the G band of graphite (i.e., about 1580 cm^−1^) confirms, once more, the coexistence of sp^3^- and sp^2^-hybridized regions, arising from the only partial reduction of GO achieved by the proposed procedure [15,25,27]. Second-order Raman features, influenced by stacking order and number of graphene sheets, can be identified as well at about 2648 cm^−1^ (2D) and 2927 cm^−1^ (D+G) [25,35]. The spectrum obtained from the analysis of Degussa P25 TiO_2_ powder is characterized by the presence of four Raman lines, which are produced by the anatase phase and can be found at about 142 (E_g_), 400 (B_1g_), 518 (superimposition of A_1g_ and B_1g_), and 639 (E_g_) cm^−1^ [25,27,34,37]. Conversely, due to its highly heterogenous composition, tionite displays a significant fluorescence over the entire spectrum, preventing the identification of any feature.

The outcome of UV-Vis spectroscopic analyses is shown in Figure S6. The rGO membrane is characterized by a strong absorption over both UV and visible ranges (Figure S6a), which is a consequence of the partial removal of oxygenated moieties and the subsequent restoration of the π-conjugated structure of the basal plane [54,55,56]. Conversely, Degussa P25 TiO_2_ exhibits a characteristic absorption edge at about 400 nm for the anatase phase [20,29]. The corresponding band gap energy (E_g_) can be determined by using the transformed Kubelka-Munk (K-M) function (Equation (3)):

(3) $${\left[F\left(R\right)\cdot h\nu \right]}^{1/n}=C\left(h\nu -E_{g}\right)$$

where “n” is a parameter depending on the type of optical transition and can be set equal to 2 in the case of indirect band gaps, such as the one of TiO_2_ [25,29,57]. By plotting the transformed K-M function versus the energy of the radiation (Figure S6b), a band gap of about 3.25 eV can be identified for Degussa P25 TiO_2_ [25,34,37]. Considering the UV-Vis spectrum of tionite powder, a clear red-shift of the absorption edge can be seen, with a corresponding reduction of the band gap to about 2.37 eV. This effect can be ascribed to the complex composition of tionite and, specifically, to the presence of iron (Table S1), which can generate impurity levels inside the forbidden band of TiO_2_ and extend its absorption to visible wavelengths as well [20,57,58,59].

### 3.2. Membranes Production and Characterization

According to the procedure reported in Section 2.2, three different membrane compositions were considered, i.e., pure rGO, rGO–TiO_2_, and rGO–TIO. The reduction time of GO was set at 24 h for all the membranes, and a 1:1 rGO–TiO_2_/–TIO weight ratio was selected for the composites, since these conditions were demonstrated as the optimum compromise to achieve good self-assembling and water cleaning capability. Membrane characteristics are summarized in Table 1. The actual weight is in line with the expected one, and the slightly lower value measured for all materials can be due to a partial material loss during manipulation. Both weight and thickness of the composite membranes increase with respect to pristine rGO, as a consequence of the insertion of either TiO_2_ or tionite.

**Figure 4:**
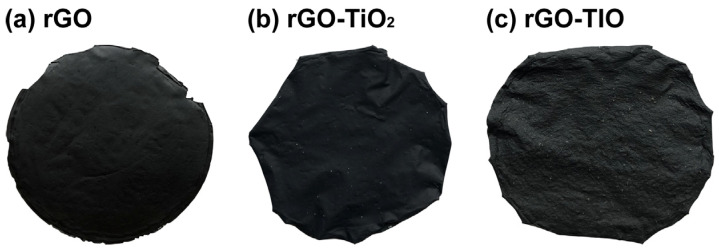
Pictures of the produced membranes: (**a**) rGO, (**b**) rGO–TiO_2_, and (**c**) rGO–TIO.

Upon visual inspection, no large differences are found between pure and mixed membranes (Figure 4). In all cases, it is evident that the adopted procedure, although consisting in the mere components’ mixture in the aqueous environment, allowed preserving the self-assembling capability of rGO and to obtain self-sustaining membranes which can be manipulated and used. Therefore, the simple production method proved to also be easily applicable to starting materials that have not undergone any refining or purification processes, such as tionite. This is particularly significant considering that the possibility to recover and reuse a waste byproduct is of paramount importance in today’s environmental and economic context, even though it is convenient only when no or very limited purification/refinement processes are required before its use.

**Figure 5:**
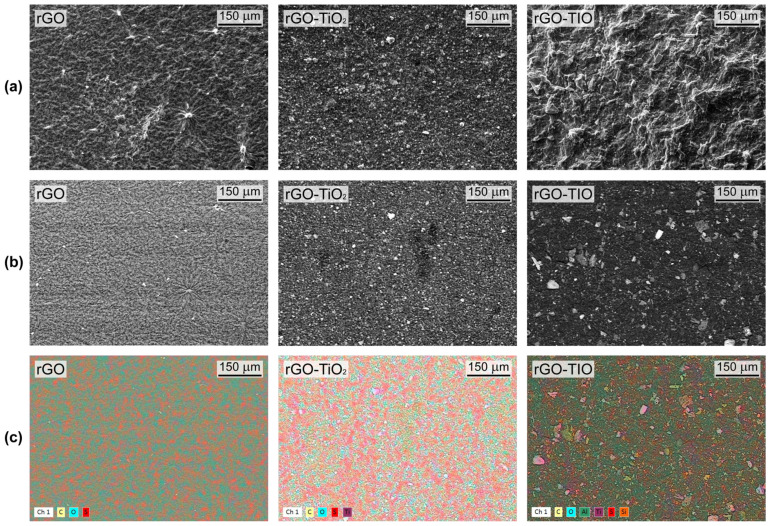
SEM pictures of pristine rGO (**left**), rGO–TiO_2_ (**middle**), and rGO–TIO (**right**) membranes: (**a**) secondary electrons, (**b**) backscattered electrons, and (**c**) elements distribution.

The morphology and composition of the membranes were tested by SEM-EDX analysis (Figure 5).

All samples exhibit a uniform surface appearance, characterized by diffuse wrinkles produced by the stacking of rGO layers in the case of rGO membranes, while rGO–TiO_2_ and rGO–TIO composites show a homogeneous distribution of several particles over their surface (Figure 5a,b). In the former, finer particulates between 1 and 6 μm of average size (as determined through ImageJ analysis) are widespread over the entire surface and can be identified with TiO_2_ agglomerates, as confirmed by compositional maps (Figure 5c) and EDX spectroscopy results (Table S2 and Figure S7a). On the other hand, the morphology of rGO–TIO appears more complicated, with a pronounced wrinkledness and the presence of larger aggregates, whose average size is between 10 and 30 μm, one order of magnitude higher than the one of TiO_2_ particles and in line with granulometric data (Figure S4). The heterogenous composition of the aggregates (Figure 5c), involving mainly Ti, Si, and Al atoms, is compatible with the one of tionite (Table S2).

**Figure 6:**
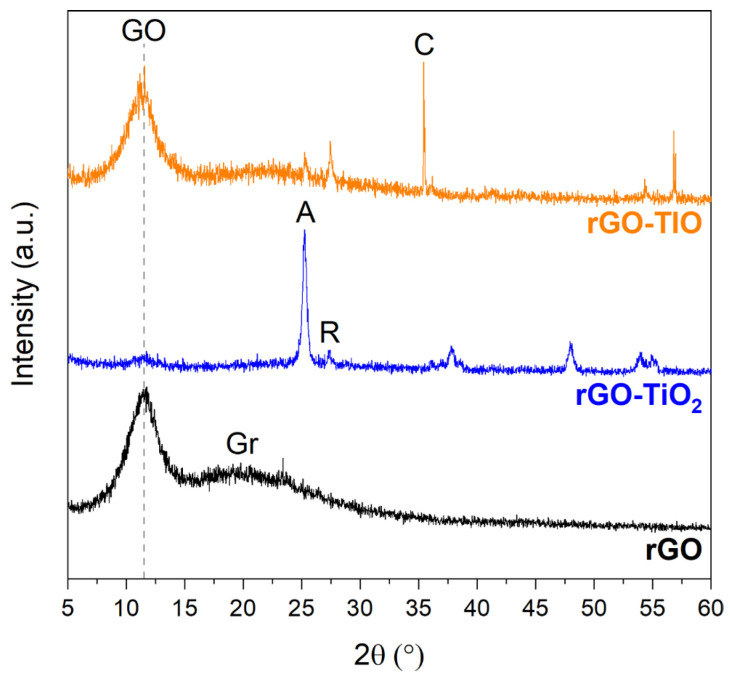
XRD patterns (bottom to top) of rGO, rGO–TiO_2_, and rGO–TIO membranes. In the figure, GO—graphene oxide, Gr—graphite, A—anatase, R—rutile, and C—calcite.

Hence, the phase composition of the membranes corresponds to a mixture of the original components, as confirmed by XRD analysis as well (Figure 6). Accordingly, in both rGO–TiO_2_ and rGO–TIO membranes, it is possible to identify the characteristic reflection at 11°, consistent with the layer spacing of GO. Other main reflections, at about 25° and 27°, correspond to the anatase and rutile phases, respectively. In the case of tionite-based membranes, the presence of calcite was also detected, confirming its presence in the membrane. By comparing the three diffractograms, it is possible to underline that the GO reflection is not affected, in either position or shape, by the combination with TiO_2_ and tionite, thus suggesting the absence of intercalation or intimate crystallite interaction with the single layers of rGO.

A further confirmation of the absence of intercalation can be found by comparing TiO_2_ crystallite dimensions of pure Degussa P25 and tionite powders, which are quite consistent with those of TiO_2_ in the corresponding rGO-based composite membranes (Table 2).

Anatase dimensions of Degussa P25 are retained in the rGO–TiO_2_ composite membrane, while a slight decrease can be observed in the same phase when tionite is mixed with rGO. On the contrary, in both composites, rutile crystallites seem to suffer a marked drop in their size with respect to the pristine materials. In the literature, this effect was ascribed to an intimate nanoscale contact between rGO and TiO_2_ nanoparticles [37]. However, such a close interaction between the components is only achievable when high purity precursors and complex preparation methods are exploited [25,26,32,35,36], which could not be selected in this work considering the desire to minimize the pretreatment of the membrane components, especially waste-derived tionite. Accordingly, it is possible to hypothesize that the preparation route proposed here may result in a lower microscale interaction between the components when compared with other membrane production procedures [26,27,30,31]. Hence, the reduction in crystallite dimensions could be partly related to a possible mild dispersion/disaggregation effect during the mixing stage, which is probably not enough to achieve an effective intercalation within single layers of rGO.

**Figure 7:**
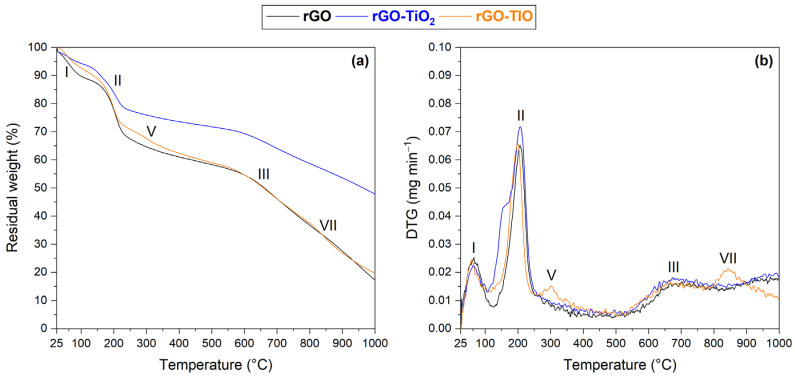
Thermogravimetric plots of rGO, rGO–TiO_2_, and rGO–TIO membranes: (**a**) residual weight and (**b**) DTG.

The reduced interaction between the Ti-containing compounds and rGO seems to be confirmed as well by the comparison of the thermograms of the membranes, as displayed in Figure 7. Indeed, the introduction of neither TiO_2_ nor tionite seems to considerably affect the thermal stability of the rGO structure and of its oxygenated functionalities since rGO characteristic weight losses (I, II, and III) remain fairly unaltered in all the investigated membranes [15,31].

Similar conclusions may be drawn by comparing the Raman spectra of the three rGO-based composite membranes (Figure S5); indeed, no evident modifications of D and G bands of rGO can be seen when either pure TiO_2_ or tionite are present. This effect might be partly explained by the absence of a close interaction between the membrane constituents, which is a direct consequence of the chosen production method (i.e., simple mixing).

Regarding the UV-Vis spectroscopic features of the composite membranes, in the case of rGO–TiO_2_, the copresence of rGO and Degussa P25 TiO_2_ seems to enhance the absorption of UV light and to induce a slight red-shift of the absorption edge with respect to pristine rGO. This effect is consistent with similar findings reported in the literature [27,28,35,37]. On the other hand, the introduction of tionite in the membrane does not seem to produce any apparent change in the absorption properties of rGO; a possible explanation may be related to the low content of anatase in tionite (about 13%), whose effect is probably hidden below the strong absorption shown by rGO.

### 3.3. Metal Capture

Metal capture is one of the membrane functions that is required for the desired final application, i.e., wastewater treatment. Therefore, it was fully investigated to evaluate if and how the adsorption functionality of rGO was preserved in the composites, especially in the case of the waste-derived TiO_2_-containing membrane (i.e., rGO–TIO). Experiments were performed by evaluating the capture capability of the different membranes towards mono-ionic Fe^3+^ and Cu^2+^ solutions, whose initial concentration was set at about 3 mg L^−1^. The results of ICP analysis, performed on the solutions after contact with the membranes, allowed to determine the amount of captured metal ions, which is reported in Table 3 in terms of retained millimoles per average membrane weight (Q_m_).

Pure rGO is able to capture both iron and copper, although a higher affinity for Fe^3+^ ions is clearly manifest, at least of one order of magnitude (0.19 and 0.05 mmol g_membrane_^−1^ for Fe and Cu, respectively), as evidenced in previous works [15]. When Degussa P25 TiO_2_ and tionite are introduced in the membrane, the adsorption capability towards both ions is preserved, with even a slight improvement in the retainment of Cu, possibly related to the presence of Ti-based compounds. Indeed, 0.11 and 0.18 mmol g_membrane_^−1^ of Fe^3+^ ions and 0.10 and 0.09 mmol g_membrane_^−1^ of Cu^2+^ ions are retained by rGO–TiO_2_ and rGO–TIO, respectively. Hence, both composite membranes still exhibit a slightly higher affinity towards Fe^3+^ ions. Moreover, it can be underlined that, despite the tionite complex phase composition (as shown in Figure 2), the ion capture capability of the rGO–TIO membrane is not affected, being very close to the one of the composite containing only pure TiO_2_.

In order to understand if the greater affinity towards Fe capture depended on the features of the sorbent or on those of the solution, chemical speciation in solution was calculated, as a function of pH, using the Hydra-Medusa software [46]. The results, displayed in Figure S8, were analyzed considering that the actual pH of iron and copper solutions was 3.7 and 6.2, respectively. Accordingly, it was found that, in the iron nitrate solution, microcrystalline and solvated Fe_2_O_3_ nanoparticles are the most probable species at pH 3.7 (Figure S8a); conversely, in the copper nitrate solution, Cu^2+^, Cu(OH)^+^, [Cu_2_(OH)_2_]^2+^, Cu(OH)_2_, and CuNO_3_^+^ could be co-present at pH 6.2 (Figure S8b). Therefore, the more efficient iron capture could result from the deposition, on the membrane surface, of the neutral species Fe_2_O_3_, already present in solution. On the contrary, the lower Cu adsorption could be explained by considering, for instance, the hindrance of Cu species, which cannot be easily allocated in the interlayers of rGO.

When Ti-containing composite membranes are applied, an additional effect could unfold, i.e., the charge repulsion between Ti and Cu ions, which are expected to all be positively charged. In this respect, the charge of the adsorbing species may play a crucial role, even if the metal capture capability of the composites is preserved in any case. However, considering the number and the complexity of the phenomena involved in the process, this point needs further investigation to be fully understood.

**Figure 8:**
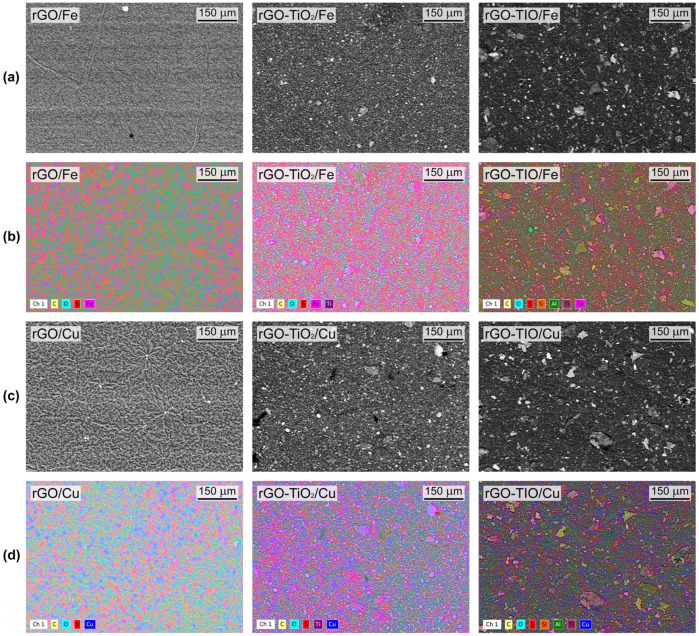
SEM pictures of rGO (**left**), rGO–TiO_2_ (**middle**), and rGO–TIO (**right**) membranes: (**a**) backscattered electrons and (**b**) elements distribution after Fe capture; (**c**) backscattered electrons and (**d**) elements distribution after Cu capture.

Then, the membranes were characterized by SEM-EDX analysis after contact with ion solutions, as reported in Figure 8, Figures S7 and S9.

By comparing SEM pictures of as-prepared membranes (Figure 5 and Figure S9a) with those taken after metal capture tests (Figure 8 and Figure S9b,c), it is worth noting that the filtration of the aqueous solution does not cause any remarkable modification of the original membranes’ morphology. This is also confirmed from the compositional point of view, as evidenced by the weight percentage of the main membrane constituents (measured by EDX spectroscopy), which does not change significantly before and after testing (Table S2 and Figure S7). Moreover, elemental maps (Figure 8b,d) clearly display the widespread presence of traces of iron and copper over the surface of all rGO-based sorbents after the corresponding capture experiments, confirming that the rGO adsorption capability is not degraded by the introduction of TiO_2_ or tionite.

EDX analyses of the membranes after metal capture are also compared with ICP-OES results in Table 4. To allow the comparison, the latter are expressed as the mass of adsorbed metal per average membrane weight. Noticeably, although obtained by two different and independent analytical methods, Fe and Cu mass percentage values from SEM-EDX and ICP-OES are remarkably close, confirming the effective ion adsorption.

### 3.4. Photodegradation

**Figure 9:**
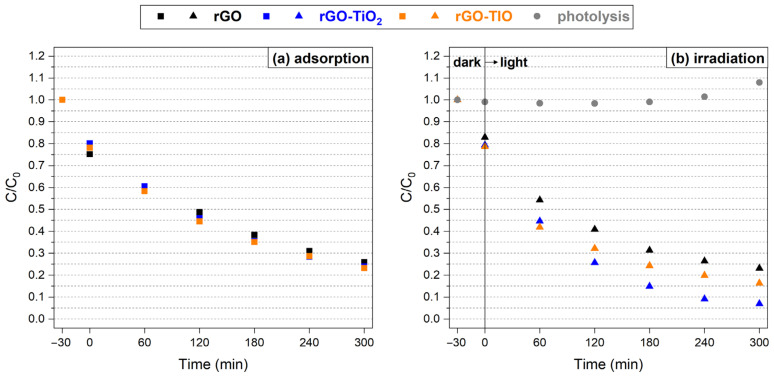
(**a**) Adsorption and (**b**) photolytic/photocatalytic degradation of Imidacloprid^®^ as a function of time and type of membrane.

The preliminary investigation of the photocatalytic properties of the rGO-based membranes led to the results displayed in Figure 9.

When tested without illumination (Figure 9a), all membranes can adsorb Imidacloprid^®^, whose concentration drops to roughly 25% of the initial value. No evident differences are recorded among the adsorption curves of the three membranes, suggesting that the capture capability is somewhat related to the rGO portion only. This feature can prove useful for the desired application since it enables almost the complete removal of the organic species from the water-based solution and, in principle, facilitates its proximity to the photoactive phase.

When the experiment is repeated under UV light irradiation (Figure 9b), a slight change in the C/C_0_ curves can be observed. The concentration drops more rapidly in the first hour of illumination and, after 5 h, the initial amount of Imidacloprid^®^ is reduced to about 23%, 7%, and 16%, respectively, for the solutions put in contact with rGO, rGO–TiO_2_, and rGO–TIO. Such a decrease can be fully ascribed to the photodegradation ability of each membrane, considering that no photolytic effects were detected in the absence of the photocatalysts.

The photocatalytic activity of the rGO-based membranes is also supported by the results of IC analyses reported in Table 5. The slight rise in the concentration of formate ions (HCO_2_**^−^**) and total residual nitrogen can be taken as a possible sign of the partial photodegradation of Imidacloprid^®^. Indeed, according to the literature, several possible intermediates can be formed during its photocatalytic reaction, depending on the operating conditions. For instance, OH^•^ and O_2_^•−^ radicals can dehydrogenate the imidazole ring or attack the nitro-amino group (R-NNO_2_) of Imidacloprid^®^, breaking the N–N bond. Moreover, the cleavage of the aromatic ring may lead to the formation of amides, short-chain linear carboxylic acids, and inorganic species such as NO_3_^−^, NH_4_^+^, and Cl^−^ [60,61,62,63].

However, considering pH and TOC values at initial (−30 min) and final (300 min) conditions, it is possible to notice that the TOC exhibits a significant increase after both adsorption and photocatalytic tests, coupled to a pH decrease. This suggests the release of some species from the membranes when immersed into the solution, possibly related to a partial material loss caused by the exfoliation of rGO layers. This behavior not only affects the individuation of decomposition products but could also be a potential drawback of the proposed system due to the potential release of additional organic compounds in the treated solutions.

**Figure 10:**
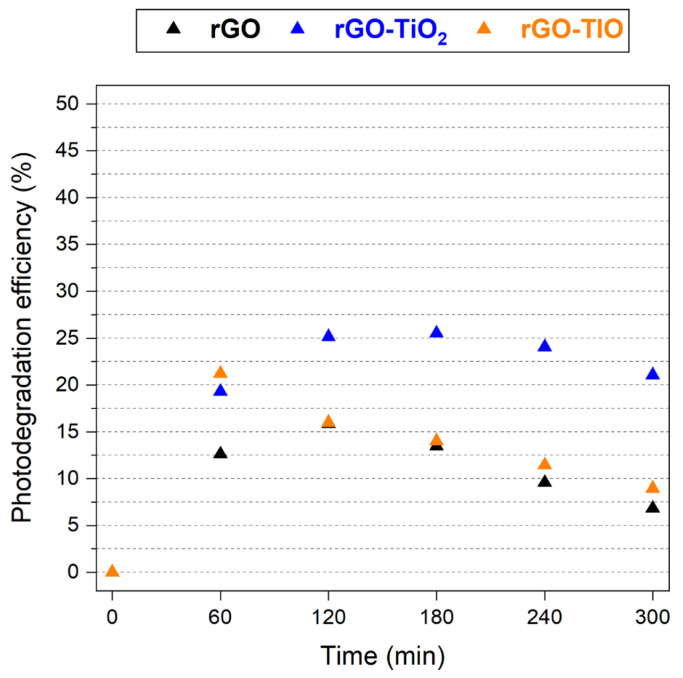
Photodegradation efficiency of rGO, rGO–TiO_2_, and rGO–TIO membranes as a function of irradiation time.

By decoupling adsorption and photodegradation components, the efficiency curves depicted in Figure 10 are obtained. The rGO, rGO–TiO_2_, and rGO–TIO membranes reach a maximum photocatalytic efficiency of about 16% (after 2 h), 25% (after 3 h), and 21% (after 1 h), respectively. The slight decrease of the photoefficiency after its maximum is likely due to a moderate evaporation of the tested solution due to UV irradiation, with a consequent small effect on the detected concentration, as visible in the photolysis curve as well (Figure 9b). Hence, it is reasonable to assume that the degradation efficiency of each membrane reaches a plateau.

The rGO–TIO composite is the fastest in achieving its efficiency peak, after only 1 h of illumination, while rGO and rGO–TiO_2_ require 2 h and 3 h, respectively. This result points out the remarkable photodegradation ability of the tionite-containing membrane, compared to the one prepared by combining rGO and pure TiO_2_. Indeed, tionite seems to possess a promising photocatalytic activity, despite its anatase content being only 13% with respect to the 83% of Degussa P25 TiO_2_, as evidenced from XRD patterns (Figure 2b,c). As discussed in Section 3.1, a possible explanation can be inferred by considering the presence of iron in the heterogeneous chemical composition of tionite. Indeed, even in the absence of strong Fe-Ti interactions, the net outcome is a narrowing of the band gap with respect to pure TiO_2_ and a more efficient absorption of visible wavelengths, with a potentially beneficial effect on the photocatalytic properties of the membrane [20,57,58,59].

Therefore, by normalizing the efficiency of the composites with respect to the actual content of anatase of rGO–TiO_2_ and rGO–TIO membranes (Equation (2)), it turns out that the latter can achieve the photodegradation of 0.16 mmol g_anatase_^−1^ after 1 h of irradiation, one order of magnitude higher and 2 h faster than the case of rGO–TiO_2_ (0.02 mmol g_anatase_^−1^ after 3 h). This remarkable result seems to confirm the feasibility of the replacement of pure commercial TiO_2_ with waste-derived tionite, even though a more thorough investigation of the photocatalytic activity of the proposed membranes should be carried out, especially focusing on a shorter time frame to better appreciate the time dependence of the phenomenon and its kinetics.

Finally, the obtained photocatalytic efficiencies are somewhat lower than the typical values reported in the literature for similar materials [64,65,66]. El-Shafai et al. [64] achieved an Imidacloprid^®^ removal efficiency of about 50% after treating for 2 h a 10^−4^ M mixed solution (methyl orange, methylene blue, and Imidacloprid^®^) with a GO@TiO_2_.ZnO.Ag hybrid nanomaterial. Behera et al. [65] used a hydrothermal method to prepare cerium-doped titania nanoparticles, deposited on reduced graphene oxide (Ce-TiO_2_/rGO), and used them as photocatalysts in a 20-mg L^−1^ Imidacloprid^®^ solution, achieving an 85% degradation after 8 h. Tismanar et al. [66] developed TiO_2_/GO thin films containing 1.4%wt of GO, which were able to decompose Imidacloprid^®^ from a 10-mg L^−1^ solution with an efficiency of about 15% after 9 h. However, it must be kept under consideration that the complex composite syntheses adopted by the previous works imply the use of highly pure materials, targeted to obtain strongly interacting nanoparticles, which can favor the photocatalytic mechanism. Moreover, most of the experiments carried out in the literature involved slurry-based or thin-film-based systems [25,26,27,28,29,31,35,36,41], which once more foster the interaction between organic contaminants and photocatalysts, as well as the exposure of the latter to the light source.

Accordingly, compared to such systems, the introduction of TiO_2_, or even of less pure tionite, in a composite membrane certainly poses more issues in terms of photoactivity. Indeed, the simple mixing of these components with rGO results in a lower particle dispersion, as well as in reduced interactions between the membrane constituents. In addition, the rGO matrix tends to incorporate the photocatalyst particles, reducing their exposure to the irradiation source. Hence, considering the very preliminary results discussed in this work, the integration of the components of the composite membranes must be further investigated and optimized to improve their photocatalytic activity.

## 4. Conclusions

This work reported the preliminary development of rGO-based composite membranes in which the self-assembling ability of rGO and the functions of the starting materials, i.e., metal capture (rGO) and photoactivity (TiO_2_), were combined, preserved, and active enough to be applied in wastewater treatment. This achievement was even more remarkable when Degussa P25 TiO_2_ was replaced with a waste-derived titania-containing material, such as tionite.

Characterization by SEM-EDX and XRD of the produced materials (rGO, rGO–TiO_2_, and rGO–TIO) allowed verifying the efficient, homogeneous mixing of the composite components and the preservation of their features. The lack of a close interaction between rGO and TiO_2_/TIO was also verified by means of Raman and UV-Vis spectroscopies, which demonstrated the absence of significant changes to the graphitic structure of rGO and to its light absorption properties.

Concerning metal capture experiments, all self-standing membranes displayed a good capability to adsorb Fe^3+^ and Cu^2+^ ions, with values of the order of 0.1–0.2 mmol g_membrane_^−1^. The ion capture ability, mostly ascribed to the rGO part of the composite, was not degraded by the insertion of Ti-based species in the membrane, and it was even slightly enhanced. A somewhat higher affinity towards iron was also recorded, resulting from the species present in the solution rather than from the adsorbent composition.

Similarly, the presence of TiO_2_ or tionite improved the photocatalytic behavior of the membranes, with a maximum degradation efficiency of 25% (after 3 h) for rGO–TiO_2_ and of 21% (after 1 h) for rGO–TIO, compared to the 16% (after 2 h) of pristine rGO. The result of tionite-containing composites is even more remarkable considering the low material purity, the large particle size, and the fact that the anatase content of tionite is roughly 1/6 of the one in commercial titanium dioxide.

Therefore, the presented results demonstrate that both functionalities of interest of the membranes (i.e., metal capture and photoactivity) are maintained without the need for specific pretreatments of the TiO_2_-containing components. This also supports the feasibility of replacing pure Degussa P25 TiO_2_ with waste-derived tionite for the manufacturing of self-standing rGO–TIO composites able to treat hybrid wastewater streams.

Future works should focus on optimizing the composition of the membranes and the integration between rGO and the TiO_2_-based constituents, so as to enhance both metal capture and photodegradation capabilities. Moreover, testing with mixed inorganic–organic solutions, simulating real wastewater, and eventually with real polluted streams should be carried out as well, in order to assess the influence of multiple species and different concentrations on the function of the material.

## Figures and Tables

**Table 1:** Weight and thickness of the produced membranes.

Membrane	Expected Weight (mg)	Actual Weight (mg)	Thickness (µm)
rGO	8	7.53 ± 0.31	11.80 ± 2.57
rGO–TiO_2_	16	15.00 ± 0.92	35.82 ± 9.43
rGO–TIO	16	12.33 ± 0.64	53.11 ± 8.01

**Table 2:** Anatase and rutile crystallite dimensions in pristine TiO_2_ and tionite, as well as in the corresponding composite membranes.

Sample	Crystallite Dimensions (nm)	
	Anatase	Rutile
TiO_2_	19	76
rGO–TiO_2_	19	36
TIO	48	101
rGO–TIO	31	60

**Table 3:** Specific captured amounts of Fe^3+^ or Cu^2+^ ions as a function of membrane weight (Q_m_, Equation (1)).

Membrane	Q_m,Fe_ (mmol g_membrane_^−1^)	Q_m,Cu_ (mmol g_membrane_^−1^)
rGO	0.19	0.05
rGO–TiO_2_	0.11	0.10
rGO–TIO	0.18	0.09

**Table 4:** Comparison of ICP-OES and SEM-EDX captured metal mass percentages.

Membrane	%wt_Fe_ ^a^ (ICP-OES)	%wt_Fe_ (SEM-EDX)	%wt_Cu_ ^a^ (ICP-OES)	%wt_Cu_ (SEM-EDX)
rGO	1.08	0.11	0.31	0.31
rGO–TiO_2_	0.62	1.31	0.63	0.72
rGO–TIO	1.02	1.22	0.56	1.18

^a^ Normalized with respect to the average membrane weight reported in Table 1.

**Table 5:** The pH, total organic carbon (TOC), and species detected by ion chromatography (IC) in the Imidacloprid^®^ solution before (−30 min) and after (300 min) testing with rGO, rGO–TiO_2_, and rGO–TIO membranes.

Test	Membrane	Time (min)	pH	TOC (mg L^−1^)	Anions ^a^ (mg L^−1^)		Residual nitrogen (mg L^−1^)			
					CH_3_COO^−^	HCO_2_^−^	NO_2_^−^	NO_3_^−^	NH_4_^+^	TOT
photolysis	-	−30	6.9	2.55	-	0.11	-	-	0.01	0.01
		300	7.0	6.29	-	0.70	0.03	-	0.11	0.14
adsorption	rGO	−30	6.1	2.25	-	-	-	-	-	-
		300	2.9	9.67	>2 (2.85)	0.16	-	-	0.05	0.05
	rGO–TiO_2_	−30	5.5	2.75	-	0.07	-	-	-	-
		300	2.6	12.80	-	0.35	-	-	0.05	0.05
	rGO–TIO	−30	6.4	2.57	-	0.08	-	-	-	-
		300	4.1	11.74	>2 (2.16)	0.20	-	-	0.01	-
photocatalysis	rGO	−30	6.9	2.61	-	0.11	0.02	-	0.01	0.03
		300	3.5	15.31	>2 (2.44)	0.77	-	0.05	0.13	0.18
	rGO–TiO_2_	−30	6.5	2.52	-	0.11	-	-	0.02	0.02
		300	3.5	12.47	1.18	1.18	0.01	0.06	0.09	0.16
	rGO–TIO	−30	6.6	2.30	-	0.11	-	-	0.01	0.01
		300	4.5	4.76	-	>2 (2.58)	-	0.06	0.07	0.13

^a^ Data in brackets are outside the calibration range of the instrument.

## Data Availability

The data presented in this study are available on request from the corresponding author.

## Supplementary Materials

The following supporting information can be downloaded at https://www.mdpi.com/article/10.3390/nano13061043/s1, Figure S1. Metal adsorption procedure, Figure S2. Structural formula of Imidacloprid^®^, Figure S3. Experimental setup of photocatalysis tests, Figure S4. Particle size and cumulative distribution curves of (a) Degussa P25 TiO_2_ and (b) tionite, Table S1. Chemical composition of tionite, as supplied by Opigeo S.r.L, Figure S5. Raman spectra of Degussa P25 TiO_2_ and tionite in powder form, as well as of rGO, rGO–TiO_2_, and rGO–TIO membranes, Figure S6. UV-Vis diffuse reflectance spectra of Degussa P25 TiO_2_ and tionite in powder form, as well as of rGO, rGO–TiO_2_, and rGO–TIO membranes: (a) absorbance versus wavelength and (b) transformed Kubelka–Munk function versus light energy, Table S2. Membranes composition as measured by EDX spectroscopy, Figure S7. EDX spectra of rGO (left), rGO-TiO_2_ (middle), and rGO–TIO (right) membranes: (a) pristine state, (b) after Fe capture, and (c) after Cu capture, Figure S8. Chemical speciation in (a) iron nitrate and (b) copper nitrate solutions, computed as a function of pH by the Hydra-Medusa software, Figure S9. Secondary-electrons SEM pictures of rGO (left), rGO–TiO_2_ (middle), and rGO–TIO (right) membranes: (a) pristine state, (b) after Fe capture, and (c) after Cu capture.
