# Supplementary material for: Reduced Graphene Oxide/Waste-Derived TiO2 Composite Membranes: Preliminary Study of a New Material for Hybrid Wastewater Treatment
Source: Nanomaterials (Basel). 2023 Mar 14;13(6):1043. doi: 10.3390/nano13061043 (PMC10055702; doi:10.3390/nano13061043)
Supplement: Supplementary file 1 [file nanomaterials-13-01043-s001.zip › nanomaterials-2253392-supplementary.pdf]

nanomaterials

Supplementary Materials

Reduced Graphene Oxide/Waste-Derived TiO_2 Composite Membranes: Preliminary Study of a New Material for Hybrid Wastewater Treatment

Andrea Basso Peressut [1,*], Cinzia Cristiani [1], Giovanni Dotelli [1,*], Anna Dotti [1], Saverio Latorrata [1], Ana Bahamonde [2], Antonio Gascó [3], Daphne Hermosilla [3] and Riccardo Balzarotti [4]

[1] Department of Chemistry, Materials and Chemical Engineering "Giulio Natta", Politecnico di Milano, Piazza Leonardo Da Vinci 32, 20133 Milan, Italy
[2] Instituto de Catálisis y Petroleoquímica, Consejo Superior de Investigaciones Científicas, Calle de Marie Curie 2, 28049 Madrid, Spain
[3] Departamento de Ingeniería y Gestión Forestal y Ambiental, Universidad Politécnica de Madrid, Calle de José Antonio Novais 10, 28040 Madrid, Spain
[4] Department of Innovative Technologies, University of Applied Sciences and Arts of Southern Switzerland, Via la Santa 1, 6962 Lugano, Switzerland
* Correspondence: andreastefano.basso@polimi.it (A.B.P.); giovanni.dotelli@polimi.it (G.D.)

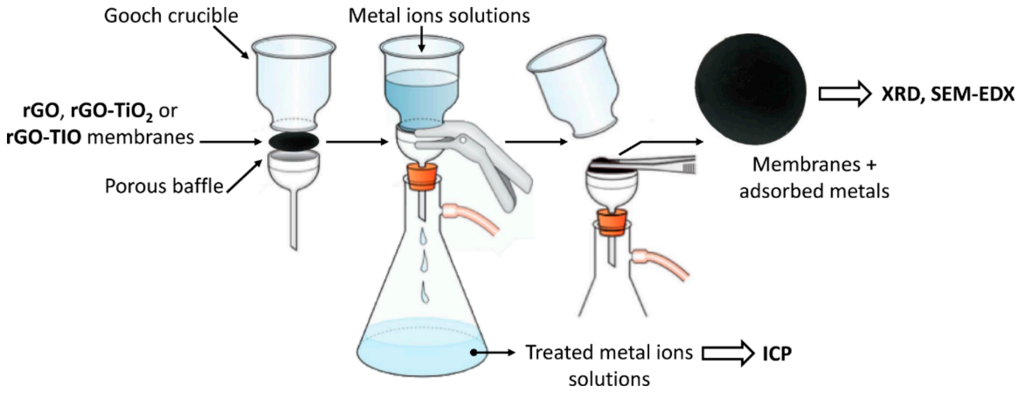

Figure S1. Metal adsorption procedure.

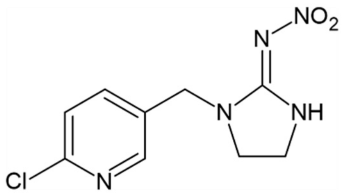

Figure S2. Structural formula of Imidacloprid®.

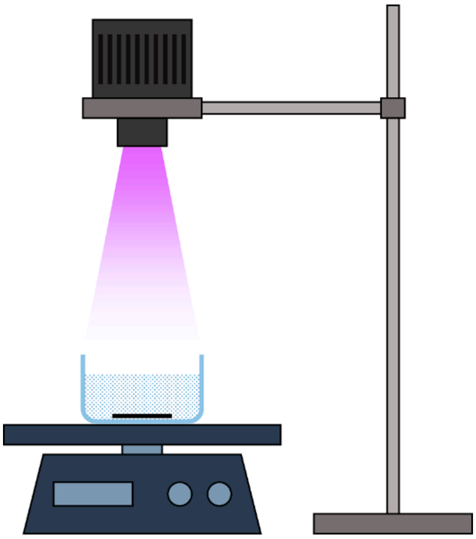

Figure S3. Experimental setup of photocatalysis tests.

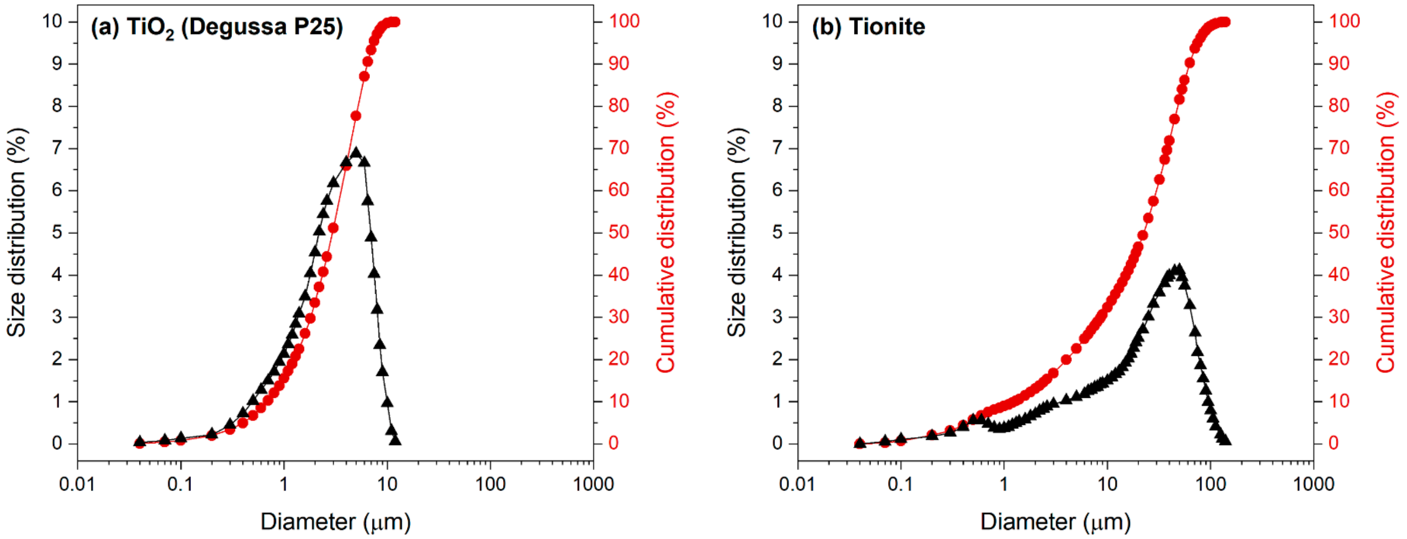

Figure S4. Particle size and cumulative distribution curves of (**a**) Degussa P25 TiO_2 and (**b**) Tionite.

Table S1. Chemical composition of Tionite, as supplied by Opigeo S.r.L.

Element	Amount (%)	Element	Amount (mg kg^{-1})
Al in Al_2O_3	2.99	As	2.30
Ca in CaO	12.70	B	44
C in CO_2	29.70	Ba	482
Fe in Fe_2O_3	2.48	Be	< 0.01
K in K_2O	0.12	Cd	< 0.01
Mg in MgO	3.00	Co	< 0.01
P in P_2O_5	< 0.01	Cu	61
Si in SiO_2	16.50	Hg	0.27
Ti in TiO_2	30.10	Mo	< 0.01
Mn in MnO	0.34	Ni	12
S in SO_3	9.30	Pb	0.93
Cr	0.01	Sb	0.75
V	0.14	Se	0.28
Cl	< 0.01	Sn	< 0.01
		Zn	60

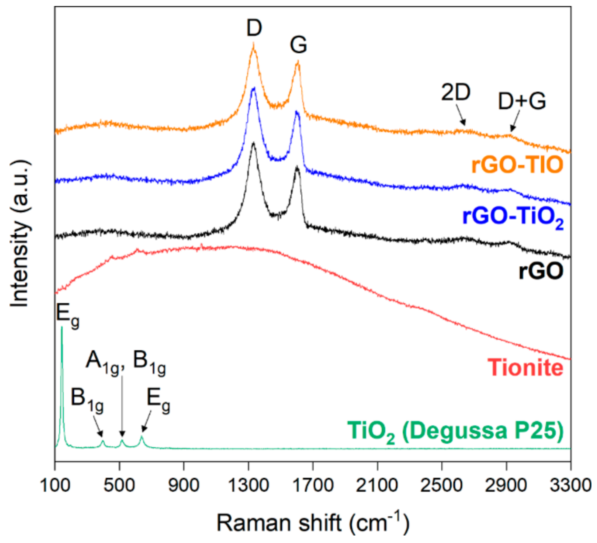

Figure S5. Raman spectra of Degussa P25 TiO_2 and Tionite in powder form, as well as of rGO, rGO–TiO_2, and rGO–TIO membranes.

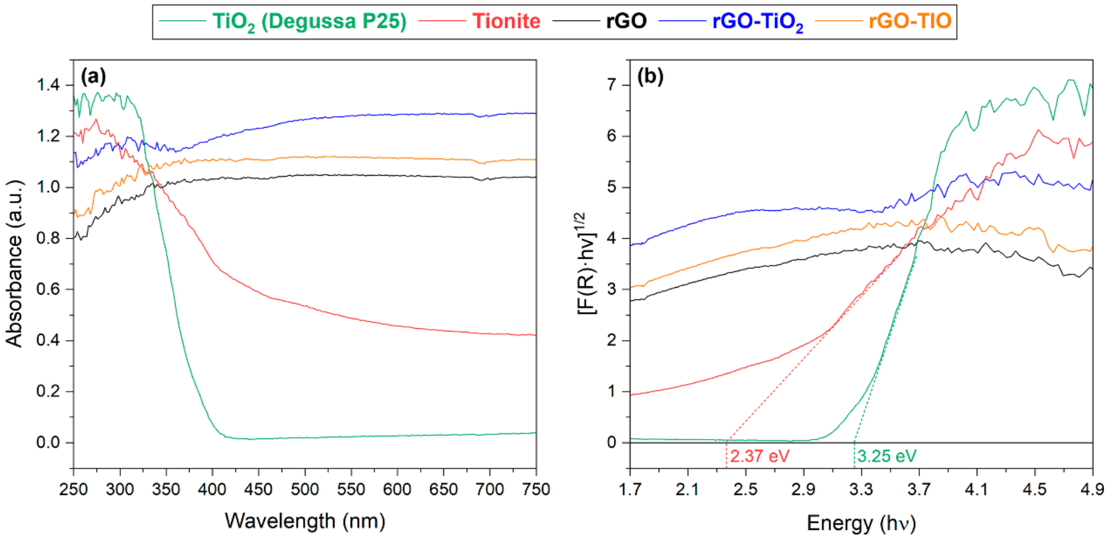

Figure S6. UV-Vis diffuse reflectance spectra of Degussa P25 TiO_2 and Tionite in powder form, as well as of rGO, rGO–TiO_2, and rGO–TIO membranes: (**a**) absorbance versus wavelength, (**b**) transformed Kubelka–Munk function versus light energy.

Table S2. Membranes composition as measured by EDX spectroscopy.

Membrane		Elements (%wt)							
		C	O	S	Ti	Al	Si	Fe	Cu
rGO	pristine	61.34	38.14	0.52	-	-	-	-	-
	after Fe	65.11	34.53	0.25	-	-	-	0.11	-
	after Cu	63.84	35.42	0.42	-	-	-	-	0.31
rGO–TiO_2	pristine	29.75	37.43	0.23	32.60	-	-	-	-
	after Fe	24.66	39.82	0.26	33.95	-	-	1.31	-
	after Cu	33.33	41.20	0.26	24.48	-	-	-	0.72
rGO–TIO	pristine	53.89	39.00	0.37	5.06	0.32	1.36	-	-
	after Fe	57.64	33.96	0.51	4.78	0.35	1.54	1.22	-
	after Cu	56.03	34.68	0.44	5.96	0.35	1.37	-	1.18

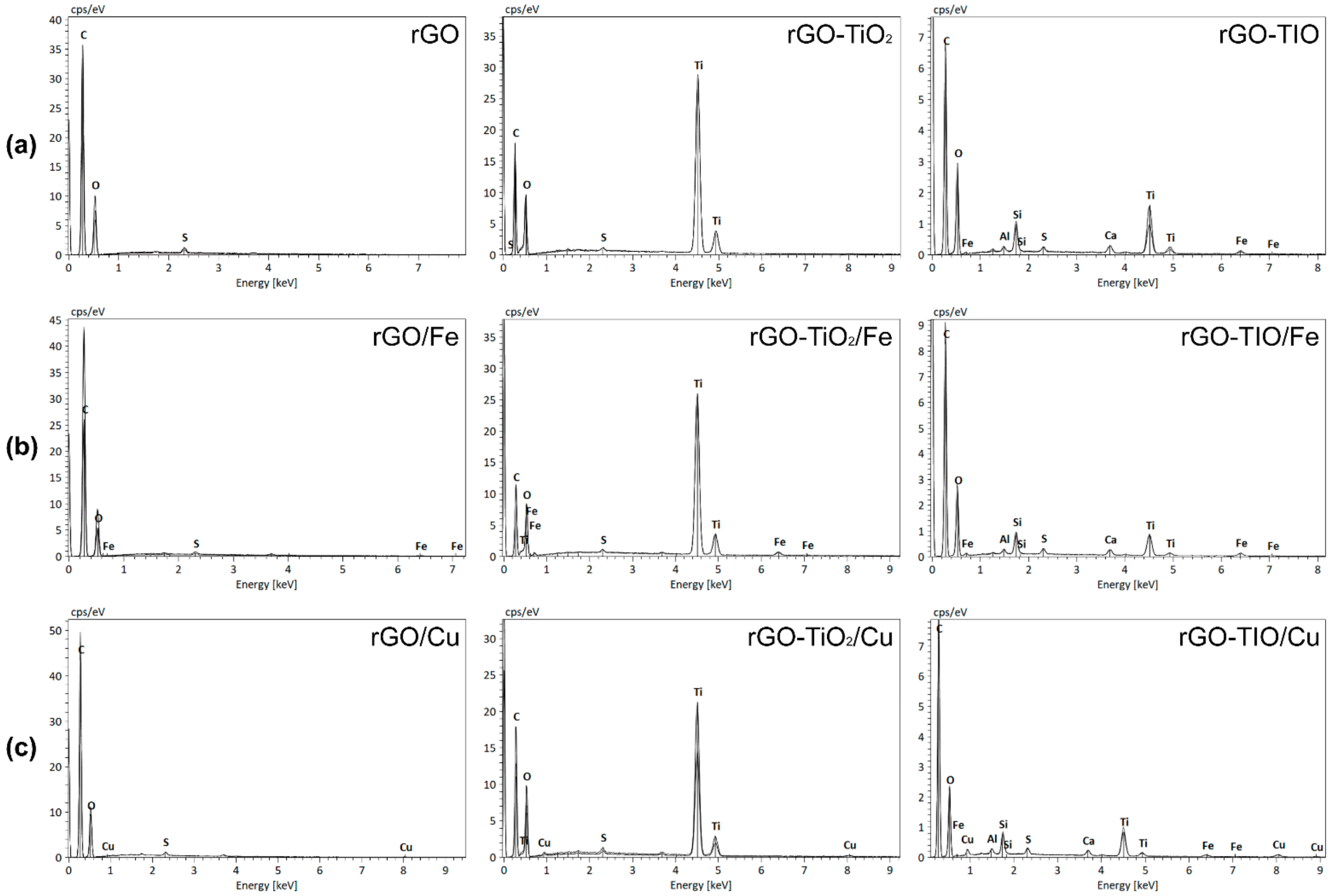

Figure S7. EDX spectra of rGO (**left**), rGO–TiO2 (**middle**), and rGO–TIO (**right**) membranes: (**a**) pristine state, (**b**) after Fe capture, and (**c**) after Cu capture.

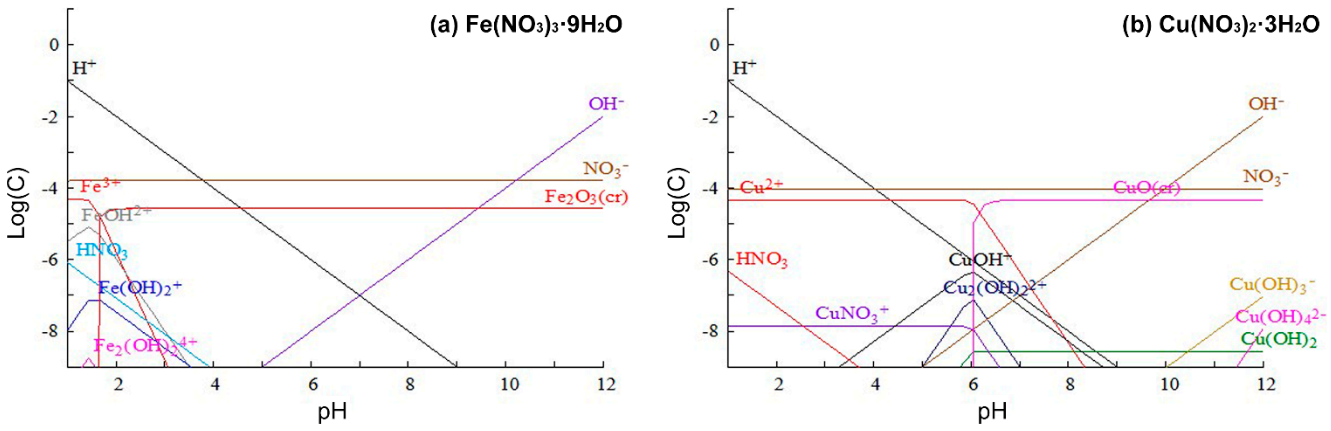

Figure S8. Chemical speciation in (**a**) iron nitrate and (**b**) copper nitrate solutions, computed as a function of pH by the Hydra-Medusa software.

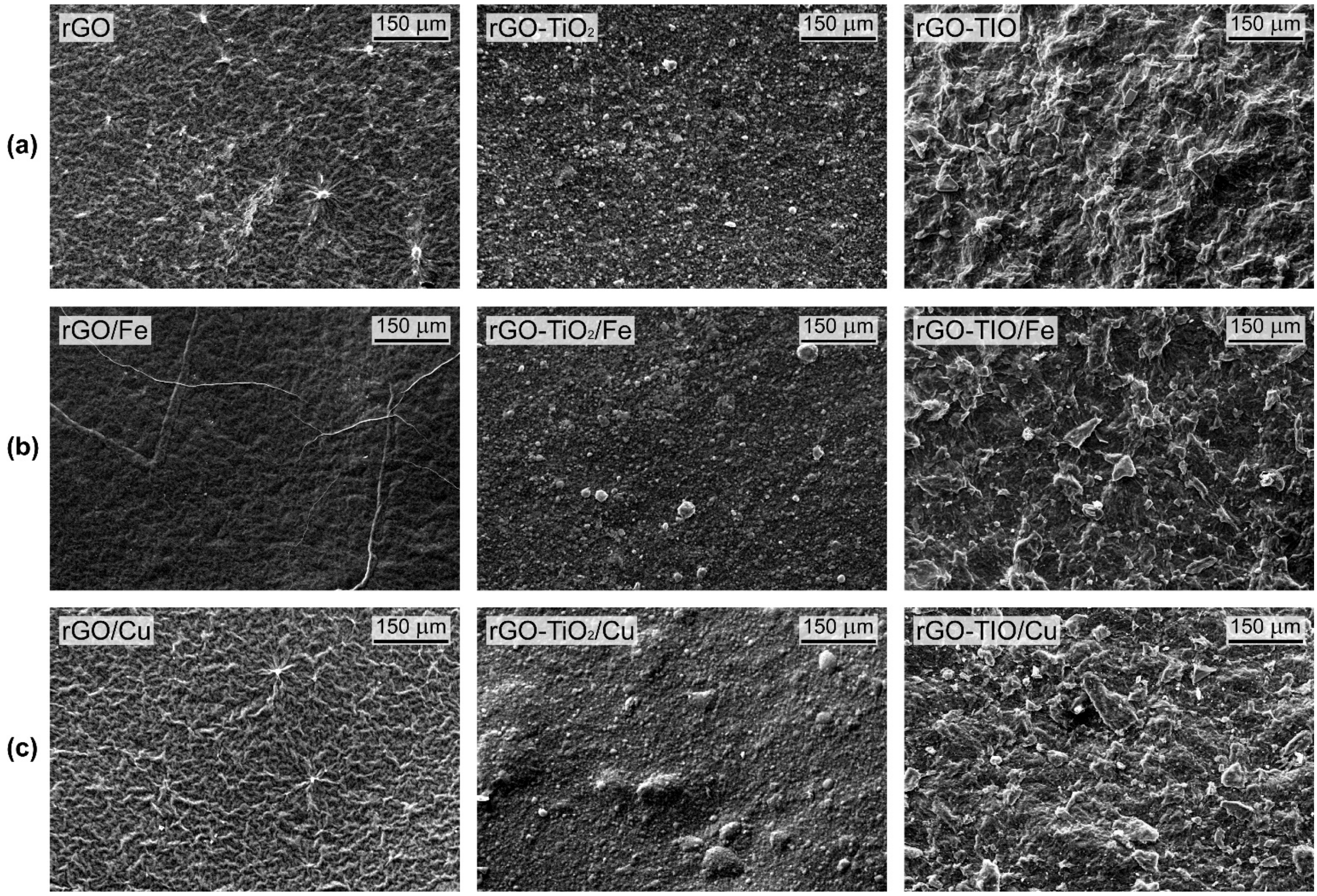

Figure S9. Secondary-electrons SEM pictures of rGO (**left**), rGO–TiO_2 (**middle**), and rGO–TIO (**right**) membranes: (**a**) pristine state, (**b**) after Fe capture, and (**c**) after Cu capture.
